# Direct administration of mesenchymal stem cell‐derived mitochondria improves cardiac function after infarction via ameliorating endothelial senescence

**DOI:** 10.1002/btm2.10365

**Published:** 2022-07-02

**Authors:** Xiaoting Liang, Yuelin Zhang, Fang Lin, Mimi Li, Xin Li, Yu Chen, Jing Liu, Qingshu Meng, Xiaoxue Ma, Enhao Wang, Lu Wei, Zhiying He, Huimin Fan, Xiaohui Zhou, Yue Ding, Zhongmin Liu

**Affiliations:** ^1^ Institute for Regenerative Medicine Shanghai East Hospital, School of Life Sciences and Technology, Tongji University Shanghai People's Republic of China; ^2^ Clinical Translational Medical Research Center Shanghai East Hospital, Tongji University School of Medicine Shanghai People's Republic of China; ^3^ Department of Emergency Medicine Guangdong Provincial People's Hospital, Guangdong Academy of Medical Sciences Guangzhou Guangdong People's Republic of China; ^4^ Department of Organ Transplantation Changzheng Hospital, Second Military Medical University Shanghai People's Republic of China; ^5^ Shanghai Engineering Research Center of Stem Cells Translational Medicine Shanghai People's Republic of China; ^6^ Department of Cardiovascular and Thoracic Surgery Shanghai East Hospital, Tongji University School of Medicine Shanghai People's Republic of China

**Keywords:** angiogenesis, endothelial senescence, mesenchymal stem cells, mitochondria transplantation, myocardial infarction

## Abstract

Mitochondrial dysfunction is considered to be a key contributor to the development of heart failure. Replacing injured mitochondria with healthy mitochondria to restore mitochondrial bioenergy in myocardium holds great promise for cardioprotection after infarction. This study aimed to investigate whether direct transplantation of exogenous mitochondria derived from mesenchymal stem cells (MSC‐mt) is beneficial and superior in protecting cardiac function in a mouse model of myocardial infarction (MI) compared to mitochondria derived from skin fibroblast (FB‐mt) and to explore the underlying mechanisms from their effects on the endothelial cells. The isolated MSC‐mt presented intact mitochondrial morphology and activity, as determined by electron microscopy, JC‐1 mitochondrial membrane potential assay, and seahorse assay. Direct injection of MSC‐mt into the peri‐infarct region in a mouse MI model enhanced blood vessel density, inhibited cardiac remodeling and apoptosis, thus improving heart function compared with FB‐mt group. The injected MSC‐mt can be tracked in the endothelial cells. In vitro, the fluorescence signal of MSC‐mt can be detected in human umbilical vein endothelial cells (HUVECs) by confocal microscopy and flow cytometry after coculture. Compared to FB‐mt, MSC‐mt more effectively protected the HUVECs from oxidative stress‐induced apoptosis and reduced mitochondrial production of reactive oxygen species. MSC‐mt presented superior capacity in inducing tube formation, enhancing SCF secretion, ATP content and cell proliferation in HUVECs compared to FB‐mt. Mechanistically, MSC‐mt administration alleviated oxidative stress‐induced endothelial senescence via activation of ERK pathway. These findings suggest that using MSCs as sources of mitochondria is feasible and that proangiogenesis could be the mechanism by which MSC‐mt transplantation attenuates MI. MSC‐mt transplantation might serve as a new therapeutic strategy for treating MI.

## 1 INTRODUCTION

Ischemic heart diseases remain the leading cause of death worldwide. Mitochondria are the power plant of the cardiomyocyte, generating more than 95% of the cardiac ATP. Complex cellular responses to myocardial infarction (MI) converge on mitochondrial malfunction, which persists and increases after ischemia, determining the extent of cellular viability and postischemic functional recovery. In a quest to ameliorate various points in the pathway from mitochondrial damage to myocardial necrosis, exhaustive pharmacologic and genetic tools have targeted various mediators of ischemia and have been used in procedural techniques without applicable success. An alternative therapeutic intervention to reverse dysfunction and restore cell normality is to transplant healthy mitochondria into the ischemic heart. The functional exogenous mitochondria will replace the harmed ones, ensuing cardioprotective functions.^1^, ^2^, ^3^

Mitochondria transplantation opens a novel horizon for treating MI. Injecting isolated viable respiration‐competent mitochondria into the ischemic zone just before reperfusion would reverse postischemic functional deterioration and suppress cellular apoptosis and limit infarct size.^4^, ^5^ The first‐in‐man pilot clinical application of autologous rectus abdominis muscle mitochondrial transplantation in pediatric patients suffering from myocardial ischemia–reperfusion injury resulted in improvement in myocardial function.^2^, ^6^ However, current mitochondria sources come mainly from autologous somatic cells or tissue, such as skeletal muscle,^7^ cardiac muscle,^4^ or pectoralis major muscle,^5^ which make harvesting mitochondria an invasive procedure for the donor and potentially raise an ethical problem that might limit clinical application of the procedure. Thus, there is an urgent need to find an alternative source of mitochondria.

Mesenchymal stem cells (MSCs) are acknowledged as efficient mitochondria donors. MSCs rescue damaged somatic cells by transferring their own healthy mitochondria through tunneling nanotubes (TNTs), microvesicles (MVs) or gap junctions, thus rescuing cellular bioenergetics. However, TNTs, MVs, and gap junctions not only mediate mitochondria exchange but also transfer microRNAs, proteins, and other organelles, such as lysosomes, making the precise characterization of the MSC mitochondrial effects on the recipient cells somewhat elusive. In a seemingly forgotten paper published in 1982, Clark and Shay described exogenously isolated mitochondria could be internalized upon simple coincubation with recipient cells.^8^ A recently developed protocol termed MitoCeption enables the validation and quantification of transferred mitochondria isolated from donor cells to recipient cells, providing a method of choice to analyze the metabolic modifications induced by exogenous mitochondria in recipient cells.^9^, ^10^

Damaged mitochondria and byproducts such as damage‐associated molecular patterns (DAMPs) are released as stress signals during cellular injury. MSCs signal these factors and elevated reactive oxygen species (ROS) levels, and enhance their bioenergetics and initiate mitochondria donation to the injured recipient cells.^11^ In a coculture system of MSCs with epithelial cells, MSCs showed a high capacity to donate their mitochondria via cytoplasmic bridges and reversed rotenone‐induced epithelial cell injuries, while smooth muscle cells and fibroblasts showed less capacity for delivering mitochondria, leading to relatively fewer therapeutic effects.^12^ In a mouse model of LPS‐induced acute lung injury, instilled MSCs formed gap junction channels within the alveolar epithelium and released mitochondria‐containing MVs that increased alveolar ATP concentrations, while fibroblasts failed to form gap junctions and rescue the cell bioenergetics in vivo.^13^ These data indicate that MSCs have the potential to serve as a source of mitochondria. However, no attempt has been made to assess the potential protective effects of MSC mitochondria against MI.

Here, we investigated whether direct transplantation of isolated MSC mitochondria is applicable and beneficial in a mouse model of MI, and we compared the therapeutic efficacy of MSC mitochondria (MSC‐mt) with fibroblast mitochondria (FB‐mt). MSC‐mt administration showed a better efficacy in promoting cardiac functional recovery and angiogenesis compared to FB‐mt in MI mice. MSC‐mt can be engulfed by endothelial cells (ECs), protecting them from oxidative challenge by suppressing apoptosis and mitochondrial ROS levels, and the protective effects were superior to FB‐mt. MSC‐mt enhanced tube formation, stem cell factor (SCF) secretion, ATP content, proliferation and angiogenesis in ECs. Mechanistically, MSC‐mt inhibited oxidative stress‐induced senescence in ECs. These findings implicate a novel strategy for MI treatment based on the concept of MSC‐mt transplantation.

## 2 RESULTS

### 2.1 Characterization of MSC and MSC‐mt

**FIGURE 1:**
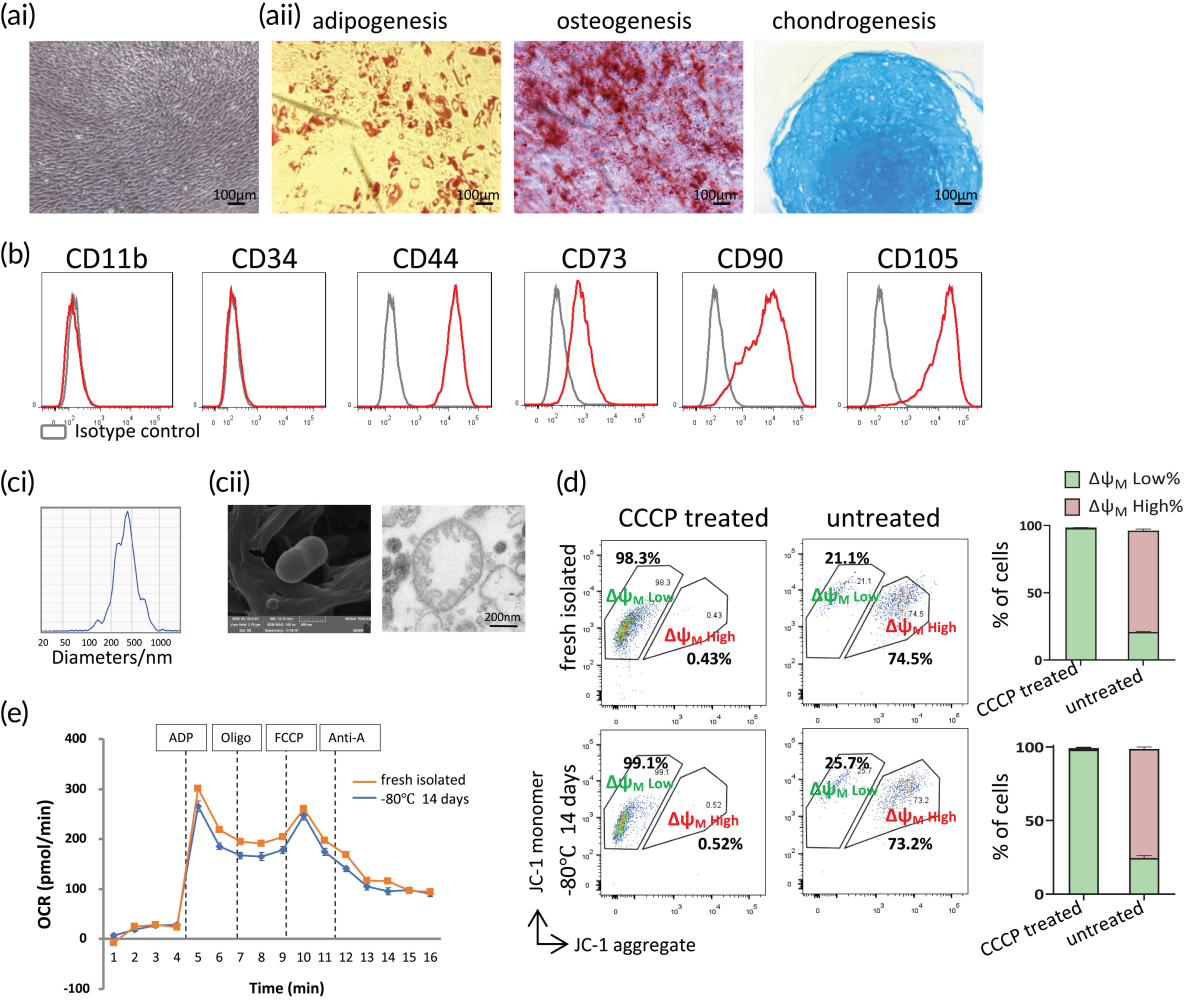
Characterization of human bone marrow‐derived mesenchymal stem cells (MSCs) and MSC‐mt. (ai) Representative fibroblast‐like morphology and (aii) assessment of the trilineage differentiation capacity of MSC. (b) Flow cytometric analysis of surface markers in MSCs. Morphological characteristics of the isolated MSC mitochondria (MSC‐mt). (ci) Representative ZetaView NTA analysis of MSC‐mt. (cii) Representative transmission electron microscopy (TEM) and scanning electron microscopy (SEM) images of isolated MSC‐mt. (d) JC‐1 staining showed that isolated MSC‐mt maintained mitochondrial membrane potential. (e) Bioenergetic activity of MSC‐mt determined by seahorse assay.

The MSC showed typical fibroblastic morphology (Figure 1ai), multidifferentiation potential (Figure 1aii), positive expression of MSC markers CD44, CD73, CD90, CD105, and negative expression of hematopoietic markers CD11b and CD34 (Figure 1b). The mean size of the isolated MSC‐mt was 469 ± 53 nm, as measured by nanoparticle tracking analysis (Figure 1ci). MSC‐mt presented a double‐layer membrane structure with mitochondria cristae and were approximately 500 nm, as observed by transmission electron microscopy (TEM) and scanning electron microscopy (SEM) (Figure 1cii). The functionality of the MSC‐mt was confirmed by JC‐1 assay. The Δψm was determined as the ratio of green (monomeric form) to red fluorescence (JC1‐aggregates). Freshly isolated MSC‐mt and stock MSC‐mt (maintained at −80°C for 14 days) had similar Δψm values (Figure 1d; Figure S1A). Treatment with the mitochondrial uncoupler Carbonyl cyanide 3‐chlorophenylhydrazone (CCCP, 20 μM for 20 min) resulted in a dramatic loss of Δψm in both the freshly isolated MSC‐mt and the stock MSC‐mt (Figure 1d; Figure S1A). Additionally, flow cytometric analysis showed equivalent particle size (SSC intensity) and granularity (FSC intensity) in freshly isolated MSC‐mt and stock MSC‐mt under normal conditions and after CCCP treatment (Figure S1B). ADP, a potent regulator of mitochondrial ATP synthesis, stimulates State III respiration. FCCP disrupts the synthesis of ATP by transporting protons across mitochondrial inner membrane. Freshly isolated MSC‐mt and stock MSC‐mt showed responses to the ADP and FCCP, as determined by the seahorse assay, suggesting MSC‐mt were bioenergetic active (Figure 1e). In general, we confirmed that the morphological and functional characteristics of the MSC‐mt, and MSC‐mt can be temporarily stored at −80°C for at least 14 days and still retained mitochondria function.

### 2.2 MSCs‐mt administration prevented cardiac function decline following infarction

**FIGURE 2:**
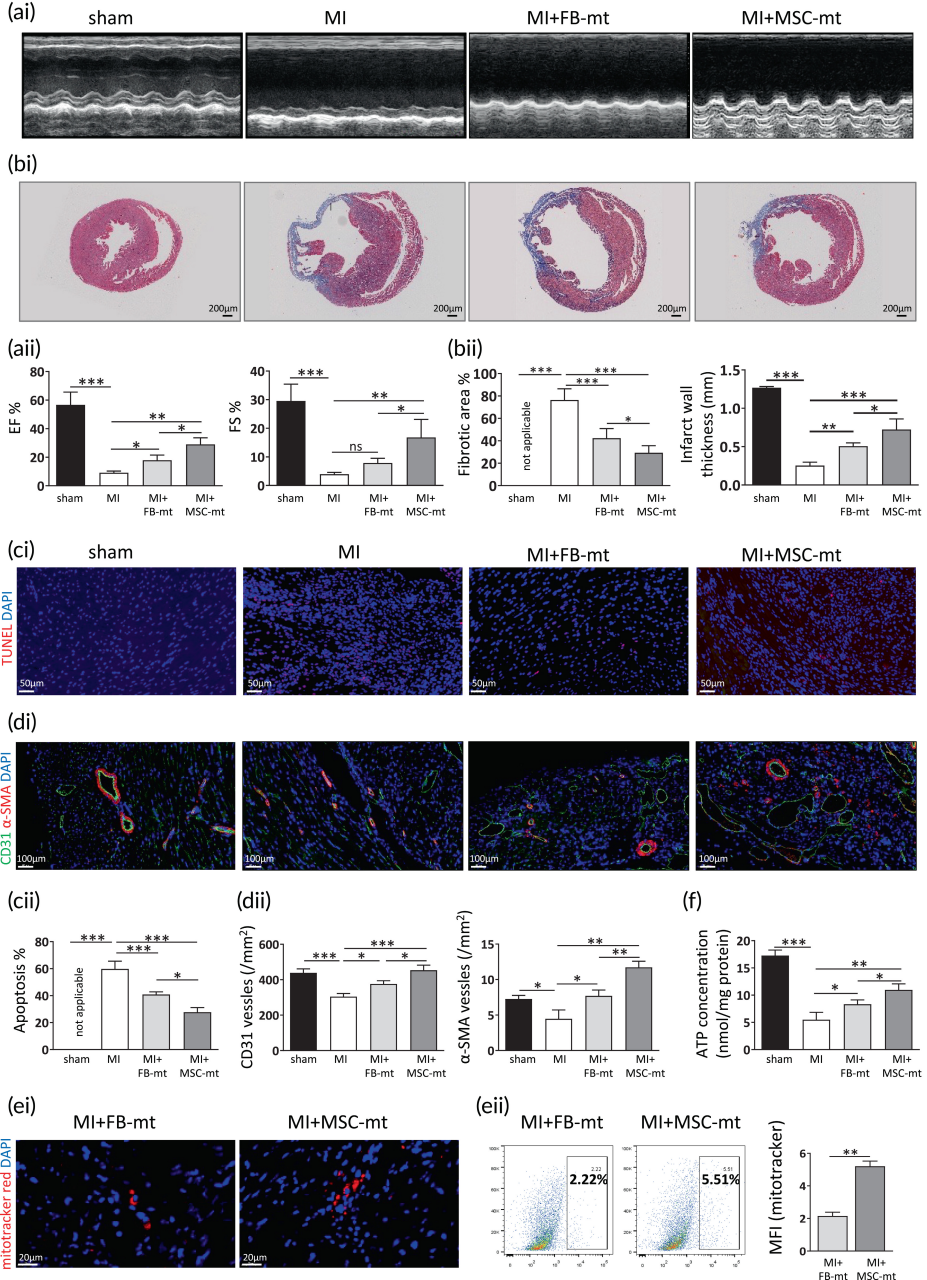
Transplantation of mesenchymal stem cells mitochondria (MSC‐mt) improved heart function following infarction. (ai) Representative images of echocardiography taken at 28 days after myocardial infarction (MI) and (aii) measurements of ejection fraction (EF) and fraction shortening (FS). *n* = 8. *ns*, not significant; *p < 0.05, **p < 0.01, and ***p < 0.001 by a one‐way analysis of variance (ANOVA) followed by Bonferroni post hoc test. (bi) Representative images of Masson's trichrome staining and (bii) results from the quantitative analysis of infarction size and infarct wall thickness. *n* = 8. *p < 0.05, **p < 0.01, and ***p < 0.001 by a one‐way ANOVA followed by Bonferroni post hoc test. (ci) Representative images of terminal deoxynucleotidyl transferase dUTP nick end labeling (TUNEL) staining and (cii) results from the quantitative analysis of apoptosis at 28 days after MI. *n* = 4. *p < 0.05, ***p < 0.001 by a one‐way ANOVA followed by Bonferroni post hoc test. (di) Representative images and (dii) results from the quantitative analysis of CD31 and α‐SMA density in the border zone of ischemic hearts. *n* = 4. *p < 0.05, **p < 0.01, and ***p < 0.001 by a one‐way ANOVA followed by Bonferroni post hoc test. (ei) Representative immunostaining and (eii) flow cytometry analysis of mitotracker red in the ischemic heart at 7 days post‐MI. *n* = 4. **p < 0.01 by an unpaired *t*‐test. (f) Measurement of ATP content in infarcted heart. *n* = 5. *p < 0.05, **p < 0.01, and ***p < 0.001 by a one‐way ANOVA followed by Bonferroni post hoc test.

Cardiac function and fibrosis were evaluated at 28 days post infarction. Transthoracic echocardiography showed significant increases in cardiac dimensions and decreases in cardiac performance in the MI group compared with the sham group (Figure 2a). There was a favorable trend toward amelioration of the cardiac indices of ejection fraction (EF) and fraction shortening (FS) in the FB‐mt‐ or MSC‐mt‐treated mice, with a more significant improvement in the MSC‐mt group compared with FB‐mt group (Figure 2a). Accordingly, Masson's trichrome staining showed the MI group presented a more extensive interstitial fibrosis than the sham group, suggesting a successful infarction was induced, and the MSC‐mt was superior to the FB‐mt in reducing the collagen volume fraction (Figure 2b). Compared with that of the sham group, the apoptosis rate was increased in the peri‐infarct region in the MI group (Figure 2c). Mitochondria transplantation greatly inhibited apoptosis, and the MSC‐mt was superior to the FB‐mt in attenuating apoptosis (Figure 2c). Capillary and arteriole density in the peri‐infarcted area was assessed by immunostaining with anti‐CD31 and α‐SMA, respectively. We observed a reduced CD31 and α‐SMA in the MI group compared with that of the sham group, and transplantation of FB‐mt or MSC‐mt significantly enhanced angiogenesis in the peri‐infarct area (Figure 2d). Notably, the MSC‐mt group had a higher CD31 and α‐SMA expression than FB‐mt group (Figure 2d). Exogeneous mitochondria were labeled by mitotracker red pretransplantation, and the fluorescent signals were detectable at 7 days after transplantation, as evidenced by immunostaining and flow cytometry (Figure 2e). Notably, the mitochondria fluorescent signal was higher in MSC‐mt group compared to FB‐mt group (Figure 2e). Mitochondria administration significantly increased myocardial ATP content in ischemic hearts at 7 days post MI, and this effect was more prominent in the mice transplanted with the MSC‐mt compared to FB‐mt (Figure 2f). To trace how long the MSC‐mt can be detected in vivo, we measured human mtDNA in the peri‐infarct myocardium used a previously described method.^14^ MSC‐mt injected myocardium showed 13 times higher human mtDNA when compared to FB‐mt group at 7 days after MI (Supplementary Figure S2). The amount of human mtDNA in both MSC‐mt and FB‐mt groups declined at 14 days postinfarction and was not detected in either group at 28 days postinfarction (Supplementary Figure S2). Collectively, these data suggested MSC‐mt transplantation facilitated cardiac function recovery through enhanced angiogenesis.

### 2.3 MSC‐mt transferred to recipient endothelial cells

**FIGURE 3:**
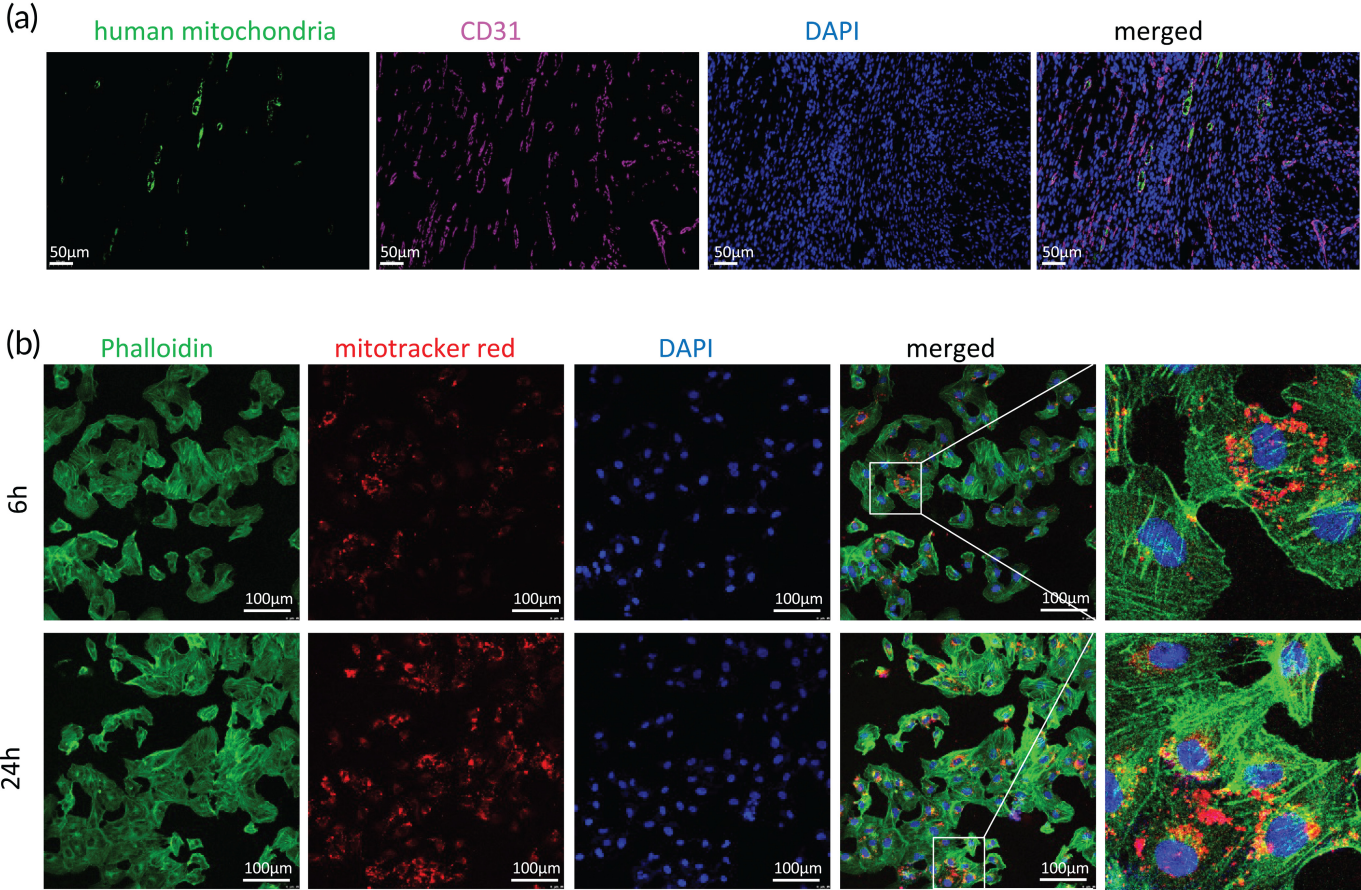
In vivo and in vitro tracking of mesenchymal stem cells mitochondria (MSC‐mt) in endothelial cells. (a) Representative immunostaining of endothelial marker CD31 and human mitochondria in the ischemic heart at 7 days post‐MI. *n* = 4. (b) Representative images of isolated MSC‐mt transferred into human umbilical vein endothelial cells (HUVECs) after 6 and 24 h of coculture. *n* = 4.

As we determined an enhanced angiogenesis in the mitochondria transplanted MI hearts, we investigated whether the endothelial cells (ECs) were able to internalize mitochondria. As MSC‐mt were derived from human, they can be tracked by anti‐human mitochondria. The fluorescent human mitochondrial signals were detectable in CD31 positive endothelial cells at 7 days post MI, suggesting the ECs were capable of internalizing MSC‐mt (Figure 3a). This phenomenon was further analyzed in vitro. The MSC‐mt were labeled by MitoTracker red, and the recipient HUVECs were labeled by phalloidin (green). After 6 and 24 h of coculture, the exogeneous MSC‐mt could be detected in the recipient HUVECs, and the fluorescence signal was higher after 24 h of co‐culture compared to 6 h of co‐culture (Figure 3b). Live‐cell imaging showed a dynamic MSC‐mt (mitotracker red labeled) transfer to HUVECs (cell trace green labeled) (Videos S1 and S2).

### 2.4 MSC‐mt protected HUVECs from oxidative stress

**FIGURE 4:**
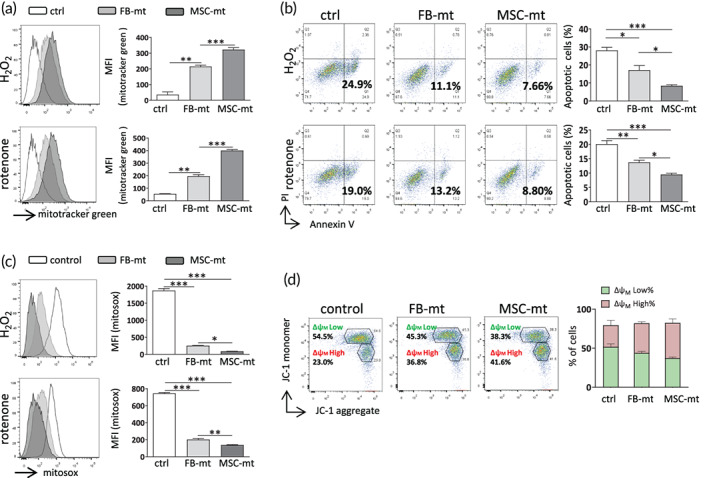
Mesenchymal stem cells mitochondria (MSC‐mt) protected human umbilical vein endothelial cells (HUVECs) from oxidative stress‐induced damage. (a) Representative flow cytometry results obtained for FB‐mt or MSC‐mt‐treated HUVECs with H_2_O_2_ or rotenone treatments. *n* = 3. **p < 0.01, ***p < 0.001 by a one‐way analysis of variance (ANOVA) followed by Bonferroni post hoc test. (b) Apoptosis was measured in FB‐mt or MSC‐mt‐treated HUVECs by Annexin V‐PI staining. *n* = 3. *p < 0.05, **p < 0.01, and ***p < 0.001 by a one‐way ANOVA followed by Bonferroni post hoc test. (c) Mitochondrial reactive oxygen species (ROS) levels in FB‐mt or MSC‐mt‐treated HUVECs were measured by MitoSOX staining. *n* = 3. *p < 0.05, **p < 0.01, and ***p < 0.001 by a one‐way ANOVA followed by Bonferroni post hoc test. (d) Mitochondrial membrane potential was assessed in FB‐mt or MSC‐mt‐treated HUVECs by JC‐1 staining. *n* = 3. MFI, mean fluorescence intensity.

To determine whether the internalized MSC‐mt exerted cytoprotective effects and to compare the effectiveness of MSC‐mt and FB‐mt, recipient HUVECs were challenged by the oxidative stress inducer H_2_O_2_ or rotenone, and then, same amount of FB‐mt or MSC‐mt were added. HUVECs were capable of internalizing FB‐mt or MSC‐mt (labeled with MitoTracker green), and a much higher fluorescence signal was observed in the MSC‐mt group compared to that in the FB‐mt group (Figure 4a). The FB‐mt and MSC‐mt prevented the HUVECs from H_2_O_2_‐ or rotenone‐induced apoptosis, and a significantly greater protective effect was observed in the MSC‐mt group compared to FB‐mt group (Figure 4b). The MSC‐mt more efficiently reduced H_2_O_2_‐ or rotenone‐induced mitochondrial ROS production compared to FB‐mt (Figure 4c). Consistent with the effects on HUVEC apoptosis, FB‐mt and MSC‐mt restored mitochondrial membrane potential reduced by H_2_O_2_ treatment, and MSC‐mt exerted a more prominent effect than FB‐mt did (Figure 4d). Collectively, these data suggested FB‐mt and MSC‐mt protected HUVECs from oxidative stress, and MSC‐mt showed a superior effect to FB‐mt.

### 2.5 Exogeneous mitochondria enhanced angiogenesis

**FIGURE 5:**
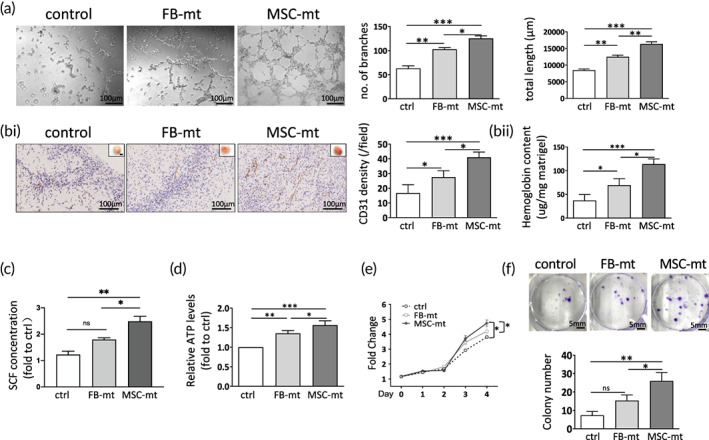
Mesenchymal stem cells mitochondria (MSC‐mt) enhanced angiogenesis in human umbilical vein endothelial cells (HUVECs). (a) Tube formation assay and quantification of FB‐mt or MSC‐mt‐treated HUVECs. *n* = 3. *p < 0.05, **p < 0.01, and ***p < 0.001 by a one‐way analysis of variance (ANOVA) followed by Bonferroni post hoc test. (bi) Representative images of the Matrigel plug (top right corner), CD31 staining and quantitative analysis of CD31 density. (bii) Measurement of hemoglobin content of the plugs. *n* = 3. *p < 0.05, ***p < 0.001 by a one‐way ANOVA followed by Bonferroni post hoc test. (c) SCF concentration in the culture supernatant as determined by ELISA. *n* = 3. *ns*, not significant; *p < 0.05, **p < 0.01 by a one‐way ANOVA followed by Bonferroni post hoc test. (d) Measurement of ATP content in FB‐mt or MSC‐mt‐treated HUVECs. *n* = 3. *p < 0.05, **p < 0.01, and ***p < 0.001 by a one‐way ANOVA followed by Bonferroni post hoc test. (e) Cell proliferation was determined by CCK‐8 assay in FB‐mt or MSC‐mt‐treated HUVECs. *n* = 3. *p < 0.05 by a one‐way ANOVA followed by Bonferroni post hoc test. (f) Colony formation assay was performed at 2 weeks after FB‐mt or MSC‐mt treatment. *n* = 3. *ns*, not significant, *p < 0.05, **p < 0.01 by a one‐way ANOVA followed by Bonferroni post hoc test.

As we observed an enhanced angiogenesis after MSC‐mt transplantation, we next examined whether exogeneous mitochondria regulates angiogenic processes. HUVECs were cocultured with FB‐mt or MSC‐mt for 4 h, and tube formation was analyzed. Marked enhancement of tube formation was observed in the FB‐mt and MSC‐mt‐treated groups, especially in the MSC‐mt‐treated HUVECs, as measured by increased branch points and total tube length (Figure 5a). An in vivo Matrigel plug angiogenesis assay was performed. A considerable number of macroscopic blood vessels was observed in the Matrigel plugs of the FB‐mt and MSC‐mt groups compared with the control group (Figure 5b). Immunostaining of the CD31 in the Matrigel plugs indicated that the FB‐mt and MSC‐mt enhanced angiogenesis (Figure 5b). The amount of hemoglobin from the plugs treated with FB‐mt or MSC‐mt was significantly higher than that found in the plugs of the control group (Figure 5b). Of note, the MSC‐mt group demonstrated a significantly greater proangiogenesis capacity than the FB‐mt group, as measured by quantification of hemoglobin content and CD31 staining (Figure 5b). SCF was reported as one of the main factors in the MSC‐mediated cardiac protection and angiogenesis.^15^, ^16^ We measured SCF concentration in the culture supernatants of control, FB‐mt‐ and MSC‐mt‐treated HUVECs, respectively. MSC‐mt treatment significantly increased the SCF concentration secretion compared to control group, while FB‐mt treatment showed no significant change on SCF secretion (Figure 5c). To further determine whether MSC‐mt affected the mitochondrial energetic function in HUVECs, ATP levels were measured. FB‐mt or MSC‐mt treatment increased the ATP levels compared to the untreated control group, and this effect was more apparent in MSC‐mt compared to FB‐mt (Figure 5d). CCK‐8 proliferation assays showed that FB‐mt treatment slightly enhanced the proliferative capacity of HUVECs, and the improvement in proliferation was more evident in the MSC‐mt‐treated HUVECs (Figure 5e). Consistently, colony formation assays confirmed that FB‐mt and MSC‐mt enhanced HUVEC proliferation and that the effect of MSC‐mt was superior to FB‐mt (Figure 5f). In the transwell setting, FB‐mt or MSC‐mt treatment did not alter the migration ability of HUVECs (Supplementary Figure S3A,C), and FB‐mt or MSC‐mt could not initiate HUVEC migration when applied in the lower chamber as attractors (Supplementary Figure S3B,C).

### 2.6 MSC‐mt ameliorated oxidative stress induced endothelial senescence via ERK pathway

**FIGURE 6:**
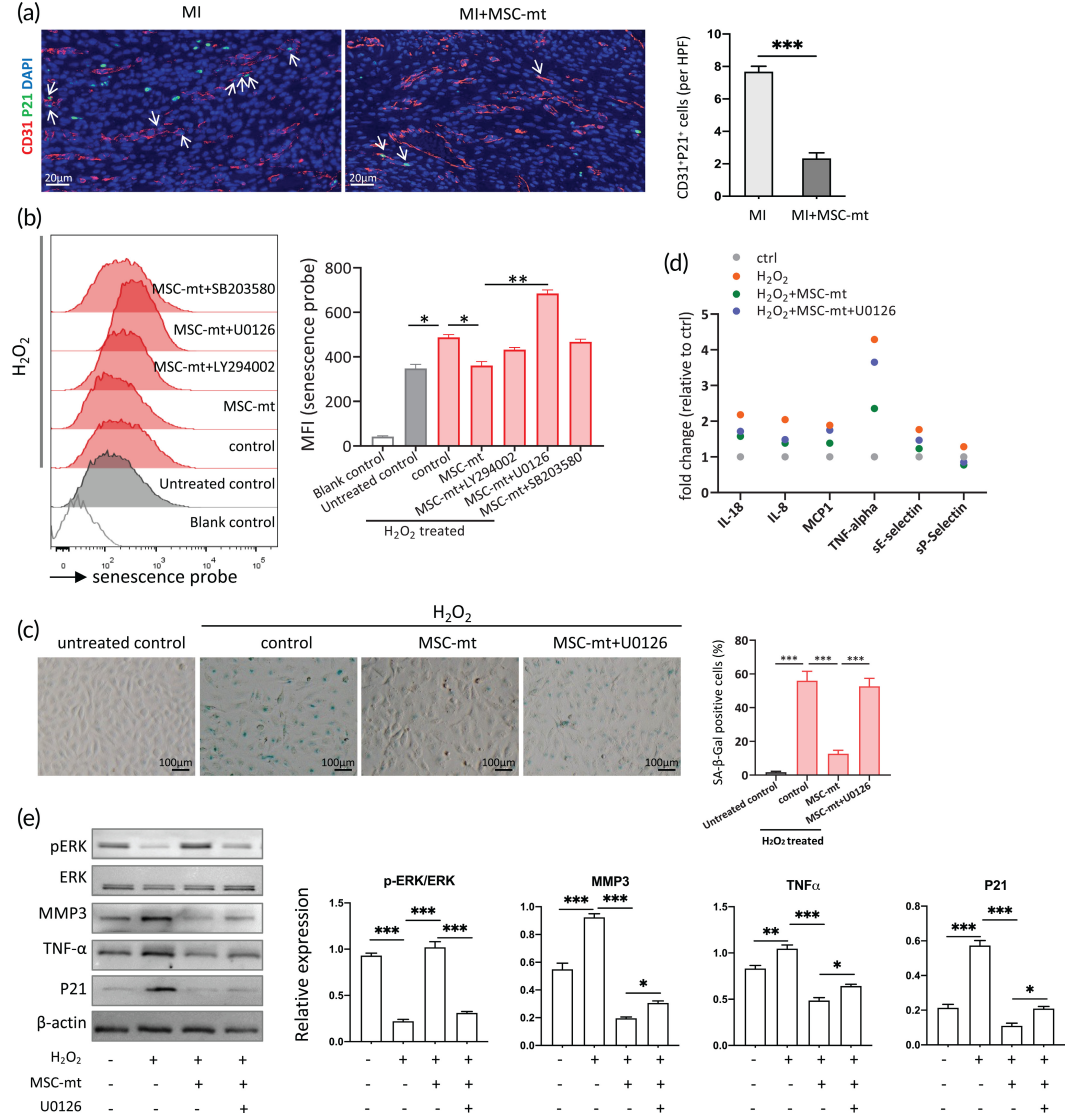
Mesenchymal stem cells mitochondria (MSC‐mt) inhibited endothelial senescence via ERK pathway. (a) Representative co‐staining images of P21 and CD31 in infarcted hearts at 7 days post‐MI. *n* = 4. ***p < 0.001 by an unpaired *t*‐test. (b) Senescent β‐Gal activity determined by flow cytometry. *n* = 3. *p < 0.05, **p < 0.01 by a one‐way analysis of variance (ANOVA) followed by Bonferroni post hoc test. (c) Representative images and quantification of SA‐β‐Gal staining. *n* = 3. ***p < 0.001 by a one‐way ANOVA followed by Bonferroni post hoc test. (d) Inflammatory cytokine expression determined by protein array. (e) Representative immunoblot images and quantitative analysis in MSC‐mt‐treated human umbilical vein endothelial cells (HUVECs) w/o pathway inhibitors. *n* = 3. *p < 0.05, **p < 0.01, and ***p < 0.001 by a one‐way ANOVA followed by Bonferroni post hoc test.

The accumulation of senescent endothelial cells impairs the function of angiogenesis and myogenesis after MI, leading to ventricular remodeling and heart function deterioration.^17^ We studied whether MSC‐mt treatment affected endothelial senescence at 7 days post‐MI. While there was no detectable P21 signal in the sham group (data not shown), the ratio of senescent marker P21 to endothelial marker CD31 significantly increased after infarction, indicating infarction‐induced senescence of the endothelial cells (Figure 6a). The ratio of P21 to CD31 decreased in the MSC‐mt group, as compared MI group, suggesting MSC‐mt treatment suppressed endothelial senescence after infarction (Figure 6a). In vitro, MSC‐mt treatment inhibited H_2_O_2_‐induced HUVEC senescence, as evidence by decreased fluorescent signal of senescence probe (Figure 6b). To examine which pathway was potentially involved in MSC‐mt regulated senescence, the LY294002 (PI3K inhibitor), U0126 (ERK inhibitor), or SB203580 (P38 inhibitor) was added to the MSC‐mt‐treated HUVECs, respectively, and cell senescence was analyzed. The result showed that U0126 treatment significantly increased HUVEC senescence, indicating the ERK pathway played an essential role in MSC‐mt regulated senescence (Figure 6b). The SA‐β‐Gal staining verified that MSC‐mt attenuated H_2_O_2_‐induced senescence, and U0126 treatment abolished this effect (Figure 6c). Senescent cells are characterized by a pro‐inflammatory senescence‐associated phenotype.^18^ We measured the pro‐inflammatory cytokine expression profile by a protein array. The result demonstrated that H_2_O_2_ induced an increased in pro‐inflammatory cytokine secretion, including IL18, IL8, MCP1, TNF‐α, sE‐selectin, and sP‐selectin (Figure 6d). MSC‐mt treatment inhibited pro‐inflammatory cytokines IL18, IL8, MCP1, TNF‐α and sE‐selectin secretion, while the addition of U0126 partially blunted this effect (Figure 6d). Accordingly, H_2_O_2_ treatment decreased p‐ERK activity, along with increased expression of senescent markers P21, MMP3, and TNF‐α (Figure 6e). MSC‐mt treatment activated p‐ERK expression and downregulate the expression of senescent markers, while U0126 suppressed these effects (Figure 6e). These data suggested MSC‐mt ameliorated oxidative stress induced endothelial senescence via ERK pathway.

## 3 DISCUSSION

**FIGURE 7:**
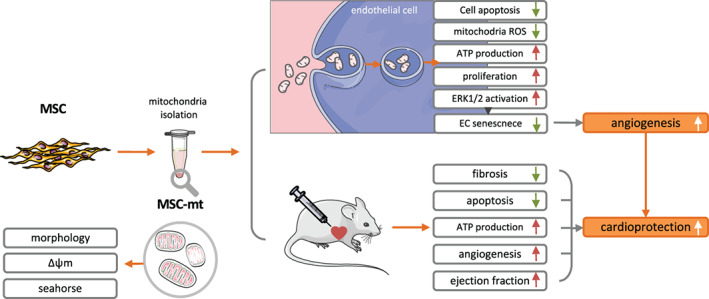
Scheme summarizing the proposed mechanism of action behind the mesenchymal stem cells mitochondria (MSC‐mt)‐mediated protective effects following myocardial infarction (MI).

The current study offers several major findings (Figure 7). First, isolated MSC‐mt presented intact membrane structure, Δψm and bioenergetics, all of which will be critical for subsequent functional applications. Next, MSC‐mt administration attenuated myocardial infarct size, increased angiogenesis and improved cardiac performance following infarction, and the protection effects was superior to FB‐mt. Third, MSC‐mt could be incorporated into recipient ECs, where they protected the ECs from apoptosis, reduced mitochondrial superoxide production, increased ATP content levels, and promoted proliferation and angiogenesis. Forth, MSC‐mt inhibited oxidative stress induced EC senescence via ERK pathway.

Proper mitochondrial function is necessary in the heart, which is of high‐energy demand. Functional abnormalities of cardiac mitochondria in MI lead to enhanced oxidative stress, diminished ATP production and energy supply and increased cell apoptosis.^19^ In 1982, internalization of exogenous mitochondria was reported by simple coincubation in vitro, without transfection reagents, medium supplements or any other interventions.^8^ However, this phenomenon has not been widely investigated, and little is known regarding the mechanisms of the mitochondria transplantation. Although the physiological relevance of this phenomenon is still under debate, the cellular uptake of exogenous mitochondria and the subsequent functional recovery of the recipient cells have been reported.^8^, ^20^, ^21^ Direct mitochondria transplantation opens a novel horizon for the treatment of cardiovascular diseases. Mitochondria isolated from pectoralis major muscle tissue were internalized by cardiomyocytes 2–8 h after transplantation and preserved myocardial energetics, maintained cell viability, and enhanced postinfarct cardiac function.^5^ In a porcine model of ischemia/reperfusion, injected mitochondria were detected after 4 weeks and were observed to effectively decrease the levels of creatine kinase (CK‐MB), cardiac troponin I (cTnI), and infarct size.^7^ In 2017, McCully et al. initiated the first clinical trial, and they reported that autologous mitochondria transplantation (isolated from nonischemic muscle tissue) improved ventricular function in five pediatric patients with ischemia/reperfusion‐associated myocardial dysfunction.^2^ Thus, the replacement of damaged mitochondria may protect the myocardium against further injury.

However, current preclinical and clinical applications of mitochondria transplantation are mostly autologous and require an immediate injection after isolation. Therefore, the quality of the isolated mitochondria could not be determined during the procedure, which could be one of the concerns in clinical application. To obtain optimal therapeutic benefits, it is important to screen and acquire mitochondria from healthy sources since isolated mitochondria may be functionally distinct depending on their origin. Emerging evidence indicates that mitochondria derived from MSCs have superior effects over those derived from mature cells. In a coculture study of MSCs with epithelial cells, mitochondria donated by MSCs effectively attenuated rotenone‐induced ROS production in epithelial mitochondria, whereas fibroblasts or smooth muscle cells were unable to deliver cytoprotection.^12^ In a mouse acute lung injury model, MSCs transferred mitochondria to the alveolar epithelium and thus increased alveolar ATP, whereas fibroblasts failed to donate mitochondria or rescue ATP production.^13^ The concept of MSC‐derived mitochondria organelle transplantation has been proposed as a revolutionary strategy for the treatment of MI,^22^ but there has been no attempt to test this proposed MI therapy to date.

The current study provides fundamental evidence for the application of mitochondria isolated from MSCs in treating MI. Isolated MSC‐mt could be temporarily stocked at −80°C and still present intact mitochondria morphology, Δψm and bioenergetics (Figure 1c–e), which enabled the quality control of isolated mitochondria before further usage. Kim et al. demonstrated that mitochondria isolated from MSCs could be transferred into various types of cells, including stem cells, cancer cells, and somatic cells, by a simple centrifugation method.^23^ They concluded that the membrane permeability of the target cells is positively correlated with the efficiency of the mitochondria transfer.^23^ Direct injection of MSC‐mt significantly decreased infarction size and enhanced angiogenesis, resulting in postischemic functional recovery (Figure 2). As we observed an enhanced angiogenesis after infarction, we suspected whether MSC‐mt have effects on ECs. We detected MSC‐mt signal in ECs in vitro and in vivo (Figure 3). Transferred mitochondria exerted biological functions in recipient cells, including restored respiratory activity and ATP production, thus delivering cellular protection under both physiological and pathological conditions.^24^, ^25^, ^26^ In accordance with previous reports, we found that internalized MSC‐mt protected HUVECs from oxidative stress‐induced damage (Figure 4). In addition, transferred MSC‐mt enhanced tube formation and angiogenesis in HUVECs by increasing ATP content and enhancing their proliferation (Figure 5).

Restoring blood supply is the foundation for the repair of ischemic diseases. Hayakawa et al. reported that mitochondria isolated from endothelial progenitor cells had upregulated angiogenesis protein expression, including bFGF, FGF‐4, plasminogen, and serpin E1, and increased tube formation in brain endothelial cells.^27^ We showed an increased blood vessel density in the ischemic hearts in the MSC‐mt‐treated mouse group compared with that of the control group, and the improvement was superior to FB‐mt (Figure 2d). These results were consistent with the in vitro study, in which MSC‐mt treatment remarkably enhanced tube formation in HUVECs (Figure 5a,b). MSC‐mt treatment significantly increased the SCF secretion, one of the main factors in the MSC‐mediated cardiac protection and angiogenesis,^15^, ^16^ while FB‐mt treatment showed no significant change on SCF secretion (Figure 5c). Increased ATP content and proliferative capacity (Figure 5d–f), but not migration (Supplementary Figure S3), might be involved in MSC‐mt‐mediated angiogenesis.

Endothelial cells comprise ~60% of the noncardiomyocytes in the heart.^28^ Endothelial cell senescence can be triggered by oxidative stress or vascular inflammation.^29^ The accumulation of senescent endothelial cells induced angiogenesis dysfunction, impairing repair after MI.^30^ Therapeutically, targeting senescent endothelial cell populations remains an attractive strategy for the treatment of cardiovascular diseases. Activating the antiaging protein Nrf2/HO‐1 prevents human endothelial cellular senescence and improves the pathological changes in cardiovascular diseases, such as thrombosis, MI, and atherosclerosis.^31^ We determined MSC‐mt treatment inhibited P21 expression in the ECs compared to MI group in vivo (Figure 6a), and MSC‐mt inhibited H_2_O_2_ induced HUVEC senescence in vitro (Figure 6b–e), indicating MSC‐mt might play a role in regulating endothelial senescence. Senescent cells are capable of affecting their microenvironment via the production of inflammatory mediators.^32^ H_2_O_2_ induced inflammatory cytokine expression in HUVECs, and MSC‐mt treatment efficiently suppressed the inflammatory cytokine expression (Figure 6d). The ERK pathway plays a dual role in regulating senescence. Experimental evidence suggests that ERK could stimulate senescence and aging, but there is also evidence that ERK is relevant to controlling senescence and aging.^33^ For instance, constitutively active expression of MEK1 (an upstream activator of ERK) in nonimmortalized intestinal epithelial cells induces cell cycle arrest, whereas it exerted an opposite role in immortalized intestinal cells, suggesting that cell type may serve a role.^34^ We investigated the role of ERK in regulating HUVEC senescence. We showed H_2_O_2_ induced a decreased pERK expression, along with an increased P21 expression and SA‐β‐GAL positive cells, suggesting ERK might play a role of anti‐senescence (Figure 6b–e). In addition, MSC‐mt treatment rescued pERK expression, along with a decreased P21 expression and SA‐β‐GAL positive cells. The ERK inhibitor U0126 abolished the effects of MSC‐mt (Figure 6–e). These data collectively suggested MSC‐mt protected ECs against oxidative stress induced senescence via activating ERK pathway.

Recently, Ikeda et al. demonstrated that mitochondria‐enriched extracellular vesicles (EVs) from human‐induced pluripotent stem cells (iPSCs)‐derived cardiomyocytes (iCMs) effectively restored energetics of ischemic myocardium.^14^ The lipid bilayer of the EVs might prevent environmental damage to the mitochondria and facilitate quick transfer of their mitochondrial cargo into the recipient cells. However, EVs delivered both mitochondrial and nonmitochondrial cargos, the latter of which contained mRNAs, noncoding RNAs and proteins clusters that initiate various intracellular signaling. Moreover, the nonmitochondrial cargos in EVs were capable to enhance the intracellular bioenergetics through activation of mitochondrial biogenesis. Thus, the therapeutic effects of EVs could not solely attributed to the mitochondria cargos, and the mechanisms of mitochondria on the recipient cells remain elusive. Second, EVs are much smaller than the expected size range of intracellular mitochondria, suggesting that most of the mitochondria contained in the EVs were relative small or fragmented. The function of these mitochondria needs further evaluation. Free mitochondrial transplantation addressed these challenges. The size of the isolated MSC‐mt ranged from 100 to 1000 nm, with a mean size of 469 ± 53 nm (Figure 1c), which is the expected size range of intracellular mitochondria. Thus, mitochondrial transplantation provides a more comprehensive assessment of the efficacy of the exogenous mitochondrial therapy. Recently, Teitell's group reported a photothermal nanoblade for transferring isolated mitochondria into somatic mammalian cells, aiming at improving the exogenous mitochondrial retention.^35^ We are looking forward to combining isolated mitochondrial and nanoengineering technologies in the future to enhance the targeting and efficiency of direct mitochondrial transplantation.

The present study has several caveats. First, the molecular mechanisms of MSC‐mt release and uptake remain unknown. Potentially, macropinocytosis and integrin‐mediated endocytosis may be involved in mitochondria internalization.^21^, ^36^ Second, although stem cells have been proposed as promising sources of healthy mitochondria, the dose of MSC‐mt should be viewed skeptically before miraculous claims trigger a wave of pop‐up clinics offering mitochondrial transplantation. In a porcine model of ischemia/reperfusion, a dose ranging from 2 × 10^5^ to 2 × 10^8^ (per gram of tissue) of mitochondria (isolated from pectoralis major muscle) injection did not result in a significant difference in decreased infarct size; therefore, 2 × 10^5^ mitochondria (per gram of tissue) was suggested as an optimal dose and has been used for initial pilot trials in humans.^6^, ^7^ In preliminary study, we had a dose‐escalating setting in the MSC‐mt transplanted group and found that dosing 3 × 10^6^ mitochondria particles per heart decreased survival rate after MI (data not shown), suggesting the safe range of mitochondria administration needs to be evaluated. Third, our data suggested that MSC‐mt can retain in the infarcted myocardium at least 14 days. Some researchers proposed that the immunogenicity of MSC‐mt could be lower than MSCs due to a lack of surface antigen expression^22^ and have shown that isolated mitochondria significantly reduce the activation of inflammation.^37^ The immunogenicity of mitochondrial transplantation needs to be vetted prior to its application in humans. Fourth, bone marrow MSCs were used in the current study. MSCs derived from primary sources and the conventional MSC manufacturing approaches are hampered by challenges with scalability and interdonor variability. Alternatively, MSCs derived from induced pluripotent stem cells (hiPSC‐MSCs) are emerging as an attractive option.^38^, ^39^ hiPSC‐MSCs showed better proliferation, survival, immunomodulatory properties and therapeutic efficacy for myocardial repair than bone marrow MSC, and possessed the capacity to transfer mitochondria to rescue mitochondrial dysfunction.^40^, ^41^ In accordance with their findings, our group showed that conditioned medium of hiPSC‐MSC (iMSC‐CdM) was superior to that derived from umbilical cord MSCs (uMSC‐CdM) in accelerating wound closure.^42^ GMP‐grade hiPSC‐MSCs have been applied in acute steroid‐resistant graft versus host disease (GVHD) in clinical trials.^43^ In the current study, bone marrow MSC‐mt showed better therapeutic efficacy compared to FB‐mt in treating MI. Based on the superiority of hiPSC‐MSCs to bone marrow MSCs, hiPSC‐MSCs can be a better mitochondria donor and represent one of the future perspectives in MSC‐mt‐based therapy. Last but not least, we showed that MSC‐mt injection enhanced angiogenesis in the infarcted heart, which inspired us to evaluate the effects of MSC‐mt on ECs; however, other cells, including immune cells, cardiomyocytes, and cardiac fibroblasts, are involved in the recovery process after infarction. The effects of MSC‐mt on these cells need to be investigated in future studies.

Taken together, the findings of the current study are the first to demonstrate the feasibility of MSC‐mt transplantation for treating MI. In contrast to MSC transplantation, mitochondrial transfer offers an “off‐the‐shelf” allogeneic therapy with few concerns of tumor formation or immune rejection. Facilitating mitochondrial transfer into the cells and investigating the potential mechanisms of protection should accelerate the application of mitochondria for the treatment of numerous clinical diseases caused by dysfunctional mitochondria.

## 4 MATERIALS AND METHODS

### 4.1 Cell culture and characterization

Human bone marrow (BM)‐MSCs were purchased from Cambrex BioScience (#PT‐2501). MSCs were routinely cultured and characterized as previously described.^44^, ^45^ The MSCs used for experiments in the current study were between passages 4 and 6. The human fibroblast (FB) cell line HFF‐1 were purchased from National Collection of Authenticated Cell Cultures (SCSP‐109) and maintained in the growth medium DMEM supplemented with 15% fetal bovine serum (FBS), with added antibiotics penicillin (100 U/ml) and streptomycin (100 μg/ml).

### 4.2 Mitochondria isolation, characterization, and labeling

Mitochondria isolation was performed using the Cell Mitochondria Isolation Kit (Life Technology, #89874) according to the manufacturer's instructions. The isolated mitochondria were dissolved in mitochondria storage buffer (MSB) (BioVision, #M1104) and stored at −80°C. For mitochondria labeling, the FB or MSCs were fluorescently labeled by staining with MitoTracker red CMXRos (Life Technology, #M7512) or MitoTracker green FM (Life Technology, #M7514) for 20 min and then subjected to mitochondria isolation. The size of mitochondria was determined by ZetaView (Particle Metrix, Germany). The morphological integrity of the mitochondria was determined by TEM and SEM as reported previously.^46^ The mass of the mitochondria was determined by Bradford assay (Bio‐Rad, #5000202). The number of mitochondria were quantified by flow cytometry as previously described.^46^ Referencing the obtained number of mitochondrial particles in 10, 20, 50, and 100 μg mitochondria determined by flow cytometry analysis, 75 μg mitochondria was interpolated to contain 1.0 × 10^6^ mitochondrial particles (data not shown). For the in vitro study, 5 μg FB‐mt or MSC‐mt was applied per 10^4^ target cells. To assess the activity of the isolated mitochondria, the mitochondrial membrane potential (Δψm) was detected using a JC‐1 assay kit (Life Technology, #M34152) according to the manufacturer's instructions. Bioenergetics of the isolated mitochondria was examined using the Seahorse XF Extracellular Flux Analyzer (Agilent Technologies, CA) as previously described.^47^, ^48^

### 4.3 MI model

Our animal study protocol conforms to the Guide for the Care and Use of Laboratory Animals published by the US National Institutes of Health (publication no. 85‐23, revised 1996) and was approved by the Institutional Animal Care and Use Committee of the Tongji University for Laboratory Animal Medicine (approval no. TJAC‐019‐142). C57/B6J mice were purchased from Shanghai Laboratory Animal Research Center (SCXK, Shanghai). Male C57/B6J mice aged 6–8 weeks were randomly divided into the following groups according to the direct intramyocardial injections administered immediately after left anterior descending artery (LAD) occlusion: direct intramyocardial injections of DMEM (MI group), FB‐mt (FB‐mt group, 0.75 × 10^6^ mitochondria particles per heart), MSC‐mt (MSC‐mt group, 0.75 × 10^6^ mitochondria particles per heart). The MSC‐mt were delivered using a 28‐gauge syringe.^5^ Control mice underwent thoracotomy without LAD ligation (sham group). Surgical procedures were performed as described previously.^49^ After echocardiographic assessment at 4 weeks, the hearts were harvested and subjected to histological examinations.

### 4.4 Echocardiographic studies

Cardiac performance was evaluated by transthoracic echocardiography (Ultramark 9, Soma Technology, Bloomfield, CT, USA) at 28 days after surgery. The dimensions were calculated using MATLAB R2011b software.

### 4.5 Morphometric evaluation of infarct size

Slides from paraffin‐embedded heart tissues were stained by Masson's trichrome to detect fibrosis among the different groups. Infarct size was quantified as the average ratio of fibrosis area to the total left ventricular (LV) area (percent fibrosis area). Infarct wall thickness was calculated by averaging three equidistant measurements of each section. Images were captured by microscopy (Leica, Germany) and analyzed with ImageJ software.

### 4.6 Immunohistochemistry

Immunohistochemical staining was performed as previously described.^44^ Briefly, the heart sections were hydrated, the antigen was retrieved, and the specimen was blocked with 5% bovine serum albumin for 30 min. Subsequently, the heart sections were stained with primary antibody at a 1:100 dilution and then incubated overnight at 4°C. The primary antibodies included anti‐CD31 (Cell Signaling Technology, #77699), α‐smooth muscle actin (α‐SMA, Abcam, #19245), human mitochondria (Abcam, #92824), and P21 (Abcam, #188224). After washing, the slides were incubated for 30 min with a fluorescent secondary antibody at room temperature. After this incubation, the slides were washed three times in phosphate buffer saline (PBS), and the slides were counterstained with 2‐(4‐Amidinophenyl)‐6‐indolecarbamidine dihydrochloride (DAPI).

### 4.7 Live‐cell imaging

For live‐cell imaging, Mitotracker red prelabeled MSC‐mt were added to HUVECs, which were labeled by cell trace green (Thermo Fisher, #C34852), and kept cultured for 24 h in an incubation chamber attached to a Leica DMi8 inverted microscope supplying 5% CO_2_ and 37°C.

### 4.8 Tissue apoptosis analysis

A terminal deoxynucleotidyl transferase dUTP nick end labeling (TUNEL) assay (Life Technology, #C10625) was utilized to measure apoptosis in vivo according to the manufacturer's instructions.

### 4.9 Mitochondrial DNA quantification

DNA was isolated using DNA isolation kit (Tiangen, #DP331). Quantification was performed by qPCR with SYBR green‐based detection (Thermo Fisher Scientific) as previously described.^14^ Briefly, relative mitochondria DNA: nuclear DNA (mtDNA:nDNA) ratio was calculated using the ΔΔCt method upon targeting mitochondrial‐encoded genes (human MT‐TL1) and nuclear‐encoded genes (mouse GAPDH). Primers used were as follows: human MT‐TL1‐F: 5′‐CACCCAAGAACAGGGTTTGT‐3′, human MT‐TL1‐R: 5′‐TGGCCATGGGTATGTTGTTA‐3′; mouse GAPDH‐F: 5′‐AACTTTGGCATTGTGGAAGG‐3′, mouse GAPDH‐R: 5′‐ACACATTGGGGGTAGGAACA‐3′.

### 4.10 Assessments of mitochondria transfer by flow cytometry and confocal imaging

For in vivo study, flow‐cytometric analysis of enzymatically digested whole heart tissue was used to analyze the MitoTracker red‐labeled FB‐mt or MSC‐mt at 7 days post‐MI. For in vitro study, MitoTracker red‐labeled MSC‐mt were added to a six‐well cell culture plate containing 1 × 10^5^ HUVECs per well, and the plate was centrifuged at 1500 g for 15 min at 4°C. To control for vital dye leakage from the isolated mitochondria, a six‐well plate containing only labeled MSCs‐mt was centrifuged at 1500 g for 15 min at 4°C. Then, the supernatant was aspirated and added to the target cells. After coculturing for 24 h, the mitochondrial transfer efficiency was monitored for quantification by flow cytometry. For confocal imaging, the HUVECs were labeled by phalloidin, and nuclei were counterstained by DAPI.

### 4.11 H_2_O_2_ and rotenone treatment

HUVECs were treated with H_2_O_2_ (1000 μM) for 1 h or rotenone (500 nM) for 2 h. Then, an appropriate amount of FB‐mt or MSC‐mt was added, and the plate was centrifuged at 1500 g for 15 min. After coculturing for 24 h, the cells were collected and subjected to downstream analysis.

### 4.12 Analysis of apoptosis, mitochondrial ROS, and mitochondrial membrane potential

Apoptosis was detected by an AnnexinV/PI apoptosis detection kit (Sony Biotechnology, #3801660) according to the manufacturer's instructions. The mitochondrial superoxide level was measured by staining the cells with MitoSOX red (Thermo Fisher, #M36008) and quantified by analyzing the fluorescence intensity through flow cytometry. The mitochondrial membrane potential (Δψm) was detected using a JC‐1 assay kit (Life Technology, #M34152) according to the manufacturer's instructions.

### 4.13 HUVEC tube formation assay

The angiogenic ability of the MSC‐mt was evaluated using the capillary tube formation assay. Briefly, HUVEC resuspended in DMEM with reduced FBS (1%) were seeded (10^4^ cells per well) in 96‐well plates coated with Matrigel (BD Biosciences, #356234). An appropriate amount of FB‐mt or MSC‐mt was added, and the plate was centrifuged at 1500 g for 15 min. After 6 h of coincubation, capillary‐like tube formation was photographed for each sample using a light microscope. The tube length and the number of branches were analyzed with ImageJ software.

### 4.14 In vivo Matrigel plug angiogenesis assay

Six‐week‐old C57/B6J mice were purchased from Shanghai Laboratory Animal Research Center (SCXK, Shanghai). A total of 1 × 10^6^ HUVECs with 50 μl MSB containing 500 μg FB‐mt or MSC‐mt and 400 μl ice‐cold Matrigel were mixed. The mixture was injected subcutaneously into the dorsal area of mice. HUVECs resuspended in the same volume of MSB and Matrigel served as the control. Fourteen days later, the Matrigel implants were harvested. Angiogenesis was analyzed by measuring the amount of hemoglobin (antibodies‐online, #ABIN1118093) and by immunostaining for CD31 (Cell Signaling Technology, #77699).

### 4.15 Enzyme‐linked immunosorbent assay

The concentration of stem cell factor (SCF) in the culture supernatants was measured using ELISA kit for SCF (Solarbio, #SEKH‐0056) according to the manufacturer's instructions.

### 4.16 ATP quantification

At 7 days post‐MI, heart tissues containing infarct and border zone was collected, lysed and quantified by BCA assay (Bio‐Rad, 5000202). The ATP levels were measured in an FB10 luminometer (Berthold Detection Systems, Germany) using an ATP bioluminescence assay kit (Beyotime, #S0026), according to the manufacturer's instructions. For in vitro ATP assessment, HUVECs were grown to 60%–70% confluency in 6‐cm plates. Then, the cells were washed with PBS, and the culture medium was exchanged for DMEM with reduced FBS (1%). Relative amounts of FB‐mt or MSC‐mt were added, and the plate was centrifuged at 1500 g for 15 min. After coculturing for 24 h, intracellular levels of ATP were measured in an FB10 luminometer (Berthold Detection Systems, Germany) using an ATP bioluminescence assay kit (Beyotime, #S0026) according to the manufacturer's instructions.

### 4.17 Cell proliferation and colony formation assay

A Cell Counting kit‐8 (CCK8) was used to measure cell proliferation. Briefly, cells were seeded onto 96‐well plates at a density of 1 × 10^3^ per well in DMEM with reduced FBS (1%). Relative amounts of FB‐mt or MSC‐mt were added, and the plate was centrifuged at 1500 g for 15 min. The cells were further incubated for additional time points (24, 48, 72, and 96 h). Ten microliters of CCK‐8 reagent (Dojindo, #CK04) was added per well and incubated for 2 h at 37°C. The absorbance was recorded at 450 nm using a 96‐well plate reader (Bio‐Rad, CA). For the colony forming assay, HUVECs were seeded onto six‐well plates at a density of 1 × 10^3^ per well in DMEM with reduced FBS (1%). Relative amounts of FB‐mt or MSC‐mt were added, and the plate was centrifuged at 1500 g for 15 min. The cells were cultured for 14 days and then fixed with 4% paraformaldehyde for 30 min and stained with 0.01% crystal violet. All assays were performed in triplicate.

### 4.18 Senescence‐associated b‐galactosidase assay

The HUVECs were cocultured with relative amounts of MSC‐mt as previously described. The inhibitors including U0126 (MedChem Express, #HY‐12031; 10 μM), or LY294002 (MedChem Express, #HY‐10108; 10 μM), or SB203580 (MedChem Express, #HY10256; 10 μM) were added and kept cultured for additional 24 h. Senescence‐associated b‐galactosidase (SA‐β‐gal) staining was performed according to the manufacturer's instructions (CellEvent Senescence Green Detection Kit, Thermo Fisher, #C10851 for flow cytometry analysis; SA‐β‐gal staining kit, Beyotime, #C0602 for cell staining).

### 4.19 Protein array

The supernatant of control, H_2_O_2_‐treated HUVECs, H_2_O_2_ + MSC‐mt‐treated HUVECs, or H_2_O_2_ + MSC‐mt + U0126‐treated HUVECs were collected and quantified by BCA assay. A total 10 μg proteins were applied for further experiment. The secretion of inflammatory cytokines was quantified by ProcartaPlex (Thermo Fisher, custom kit) according to the instructions.

### 4.20 Western blotting

The proteins in the cell lysates were quantified by BCA assay (Bio‐Rad, 5000202). Western blotting was performed using a standard protocol as previously described.^50^ The following antibodies were used: p‐ERK (Cell Signaling Technology, #4370), ERK (Cell Signaling Technology, #4695), MMP3 (Cell Signaling Technology, #14351), TNFα (Cell Signaling Technology, #6945), P21 (Cell Signaling Technology, # 2947) and anti‐β‐actin (Proteintech, #66009‐1‐Ig).

### 4.21 Statistical analysis

The values are expressed as the mean ± standard error of the mean (SEM). Comparisons between two groups were analyzed using Student's *t*‐test. Statistical differences among more than two groups were assessed by one‐way analysis of variance (ANOVA) with the Bonferroni post hoc test. A value of *p* < 0.05 was considered statistically significant.

## AUTHOR CONTRIBUTIONS

**Xiaoting Liang**: Conceptualization, Methodology, Validation, Formal analysis, Investigation, Visualization, Data Curation, Writing – Original Draft, Writing – Review and Editing, Project administration, Funding acquisition; **Yuelin Zhang**: Methodology, Validation, Formal analysis, Investigation, Visualization, Data Curation, Writing – Review and Editing; **Fang Lin**: Methodology, Validation, Formal analysis, Writing – Original Draft; **Mimi Li**: Methodology, Formal analysis, Investigation; **Xin Li**: Resources; Yu Chen: Methodology, Formal analysis, Writing – Original Draft; Jing Liu: Methodology, Formal analysis; **Qingshu Meng**: Resources; **Xiaoxue Ma**: Methodology, Formal analysis; **Enhao Wang**: Methodology; **Lu Wei**: Methodology; **Zhiying He**: Resources, Funding acquisition; **Huimin Fan**: Resources; **Xiaohui Zhou**: Resources, Funding acquisition; **Yue Ding**: Conceptualization, Writing – Original Draft, Writing – Review and Editing, Project administration, Visualization, Data Curation, Funding acquisition, Supervision; **Zhongmin Liu**: Writing – Review and Editing, Project administration, Funding acquisition, Supervision.

## FUNDING INFORMATION

This research was supported in part by the National Natural Science Grant of China (81500207 to Xiaoting Liang; 81700259 to Yuelin Zhang; and 81670458 to Xiaohui Zhou), the Pyramid Talent Project (YQ677 to Yue Ding), the Science and Technology Commission of Shanghai Municipality (17431906600), Shanghai Engineering Research Center of Artificial Heart and Heart Failure Medicine (no. 19DZ2251000), Shanghai Engineering Research Center of Stem Cells Translational Medicine (20DZ2255100), Major Program of Development Fund for Shanghai Zhangjiang National Innovation Demonstration Zone (grant nos. ZJ2018‐ZD‐004) and Peak Disciplines (Type IV) of Institutions of Higher Learning in Shanghai.

## CONFLICT OF INTEREST

The authors declare no conflicts of interest.

### PEER REVIEW

The peer review history for this article is available at https://publons.com/publon/10.1002/btm2.10365.

## Supporting information


**Supplementary Figure 1** Isolated MSC‐mt reacted to CCCP treatment. (A) JC‐1 staining showed that CCCP treatment resulted in a dramatic loss of Δψm in MSC‐mt stored at −80°C for 14 days. (B) Relative size (FSC) and internal complexity (SSC) of the MSC‐mt were determined by flow cytometry. FSC, forward scatter; SSC, side scatter; AU, arbitrary units. *ns*, not significant, *p < 0.05, ***p < 0.001 by an unpaired t‐test.
**Supplementary Figure 2.** Human‐derived mtDNA was measured in mice myocardium at Day 7, Day 14, and Day 28 by PCR; shown as fold change relative to FB‐mt‐treated myocardium. *n* = 3. *p < 0.05, **p < 0.01 by an unpaired *t*‐test.
**Supplementary Figure 3.** FB‐mt and MSC‐mt treatment did not alter the migration ability of the HUVECs either used as a motivator (A) or an attractant (B). (C) Results from the quantitative analysis of the migrated cells. *n* = 3. *ns*, not significant by a one‐way ANOVA followed by Bonferroni post hoc test.

**supplementary video 1** Live‐cell imaging showed a dynamic MSC‐mt (mitotracker red labeled) transfer to HUVECs (cell trace green labeled) in coculture system.

**supplementary video 2** Live‐cell imaging showed a dynamic MSC‐mt (mitotracker red labeled) transfer to HUVECs (cell trace green labeled) in coculture system.

## Data Availability

The datasets generated during and/or analysed during the current study are available from the corresponding author on reasonable request.
