# Supplementary material for: Direct administration of mesenchymal stem cell‐derived mitochondria improves cardiac function after infarction via ameliorating endothelial senescence
Source: Bioeng Transl Med. 2022 Jul 2;8(1):e10365. doi: 10.1002/btm2.10365 (PMC9842017; doi:10.1002/btm2.10365)

**Direct administration of mesenchymal stem cell-derived mitochondria improves cardiac function after infarction via ameliorating endothelial senescence**

Xiaoting Liang^1,2,#^, , Yuelin Zhang^3,#^, Fang Lin^2^, Mimi Li,^2^ Xin Li^3^, Yu Chen^4^, Jing Liu^1,2^, Qingshu Meng^2^, Xiaoxue Ma^2^, Enhao Wang^2^, Lu Wei^2^, Zhiying He^1,6^, Huimin Fan^2^, Xiaohui Zhou^2^, Yue Ding^4,*^, Zhongmin Liu^1,2,5,*^

^1^Institute for Regenerative Medicine, Shanghai East Hospital, School of Life Sciences and Technology, Tongji University, Shanghai 200120, P.R. China;

^2^Clinical Translational Medical Research Center, Shanghai East Hospital, Tongji University School of Medicine, Shanghai, P.R.China;

^3^Department of Emergency Medicine, Guangdong Provincial People’s Hospital, Guangdong Academy of Medical Sciences, Guangzhou, 510080, Guangdong, P.R.China;

^4^Department of Organ Transplantation, Changzheng Hospital, Second Military Medical University, Shanghai, P.R.China;

^5^Department of Cardiovascular and Thoracic Surgery, Shanghai East Hospital, Tongji University School of Medicine, Shanghai, P.R.China.

^6^Shanghai Engineering Research Center of Stem Cells Translational Medicine, Shanghai, P.R.China.

^#^These authors contributed equally to the work.

^*^Correspondence: Dr. Yue Ding, Department of Organ Transplantation, Changzheng Hospital, Second Military Medical University and Prof. Zhongmin Liu, Clinical Translational Medical Research Center, Shanghai East Hospital, Tongji University School of Medicine, Shanghai, P.R.China

Email: [dingnashi873321@163.com](mailto:dingnashi873321@163.com) (Yue Ding); [liu.zhongmin@tongji.edu.cn](mailto:liu.zhongmin@tongji.edu.cn) (Zhongmin Liu)

**supplementary information**

**supplementary Figure 1.** Isolated MSC-mt reacted to CCCP treatment. (A) JC-1 staining showed that CCCP treatment resulted in a dramatic loss of Δψm in MSC-mt stored at -80°C for 14 days. (B) Relative size (FSC) and internal complexity (SSC) of the MSC-mt were determined by ﬂow cytometry. FSC, forward scatter; SSC, side scatter; AU, arbitrary units. *ns*, not significant, **p*<0.05, ****p*<0.001 by an unpaired t-test.

**supplementary Figure 2.** Human-derived mtDNA was measured in mice myocardium at day 7, day 14 and day 28 by PCR; shown as fold change relative to FB-mt treated myocardium. n=3. **p*<0.05, ***p*<0.01 by an unpaired t-test.

**supplementary Figure 3.** FB-mt and MSC-mt treatment did not alter the migration ability of the HUVECs either used as a motivator (A) or an attractant (B). (C) Results from the quantitative analysis of the migrated cells. n=3. *ns*, not significant by a one-way ANOVA followed by Bonferroni post hoc test.

**supplementary video 1&2.** Live cell imaging showed a dynamic MSC-mt (mitotracker red labeled) transfer to Huvecs (cell trace green labeled) in coculture system.

**
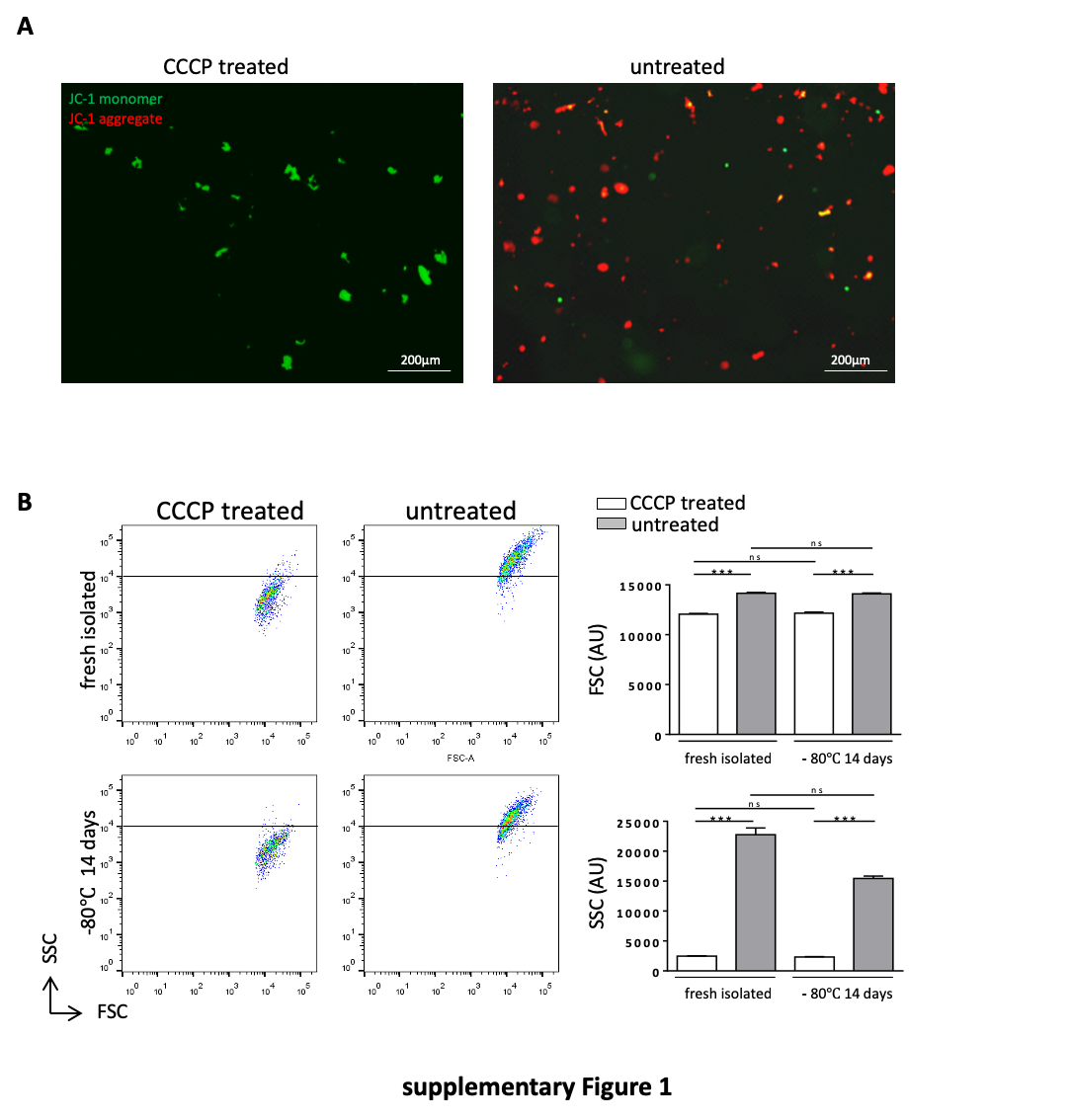
**


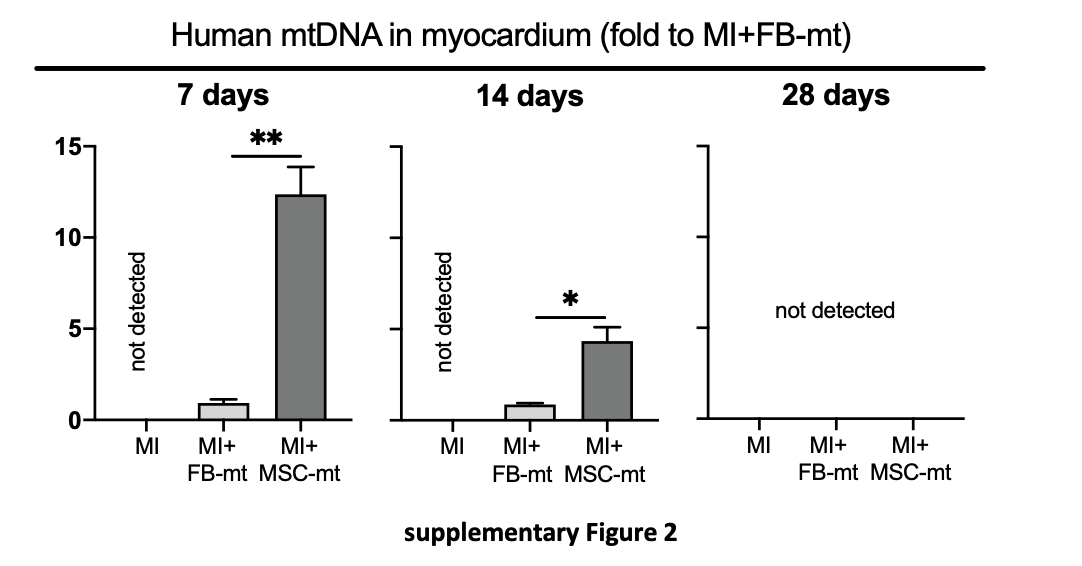


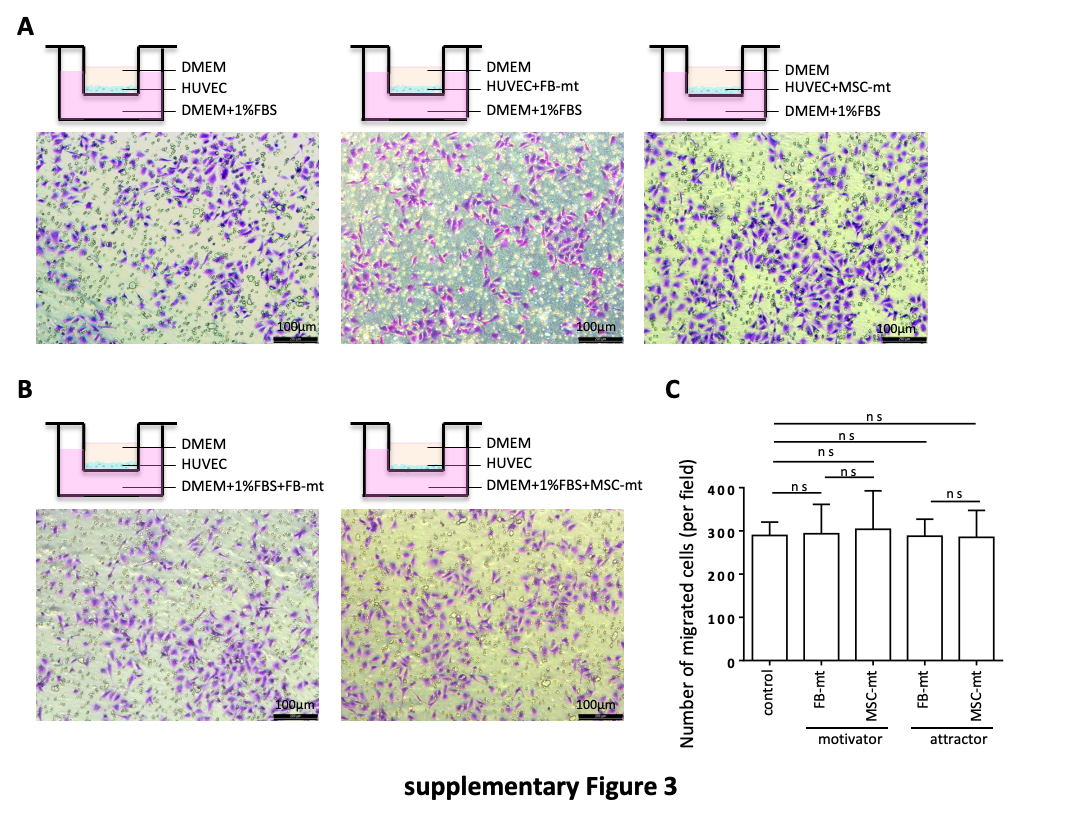

Supplement: Supplementary file 1 — Supplementary Figure 1 Isolated MSC‐mt reacted to CCCP treatment. (A) JC‐1 staining showed that CCCP treatment resulted in a dramatic loss of Δψm in MSC‐mt stored at −80°C for 14 days. (B) Relative size (FSC) and internal complexity (SSC) of the MSC‐mt were determined by flow cytometry. FSC, forward scatter; SSC, side scatter; AU, arbitrary units. ns, not significant, *p < 0.05, ***p < 0.001 by an unpaired t‐test. Supplementary Figure 2. Human‐derived mtDNA was measured in mice myocardium at Day 7, Day 14, and Day 28 by PCR; shown as fold change relative to FB‐mt‐treated myocardium. n = 3. *p < 0.05, **p < 0.01 by an unpaired t‐test. Supplementary Figure 3. FB‐mt and MSC‐mt treatment did not alter the migration ability of the HUVECs either used as a motivator (A) or an attractant (B). (C) Results from the quantitative analysis of the migrated cells. n = 3. ns, not significant by a one‐way ANOVA followed by Bonferroni post hoc test. [file BTM2-8-e10365-s002.docx]
